# Central neurocytomas: Decision-making process of postoperative treatment. Lesson learned

**DOI:** 10.1007/s10143-026-04441-4

**Published:** 2026-09-21

**Authors:** Sergio Corvino, Giuseppe Corazzelli, Giuseppe Di Nuzzo, Vincenzo Seneca, Roberto Granata, Danilo De Paulis, Mario Cella, Roberto Altieri, Giuseppe Catapano

**Affiliations:** 1 Department of Neurosurgery, P.O, “Ospedale del Mare”, Naples, 80147 Italy; 2 Department of Human Neurosciences, “La Sapienza” University of Rome, Rome, Italy; 3 Department of Neurosurgery, “Santa Maria delle Grazie” Hospital, Pozzuoli, Italy; 4 Department of Intensive Care, P.O, “Ospedale del Mare”, Naples, 80147 Italy; 5 Multidisciplinary Department of Medical-Surgical and Dental Specialties, University of Campania “L. Vanvitelli”, Naples, 80131 Italy

**Keywords:** Central neurocytomas, Cerebral ventricles, Brain tumors, Ki67, Adjuvant radiotherapy

## Abstract

Central neurocytoma is a rare intraventricular tumor with favorable prognosis following gross total resection (GTR). However, the role of Ki67-MIB1 values in the postoperative decision making-process of treatment, as well as the indication for adjuvant radiotherapy, remain debated. Medical record data of patients with confirmed diagnosis of central neurocytoma who underwent surgical resection at Department of Neurosurgery of “Ospedale del Mare” in Naples – Italy, between January and December 2022, were retrospectively reviewed. Inclusion criteria were adult patients (> 18 years old), minimum follow up 42 months, complete demographic, clinical, neuroradiological and histopathological data, type of management, complications and outcome, time to recurrence, follow-up duration. Five adult patients were enrolled according to the inclusion criteria. Most patients were women (80%), with mean age 24 years old. Main presenting symptoms were headache and visual deficit. Lesions always involved lateral ventricles, with extension to the third ventricle in all but one cases. Transcortical approach to the ipsilateral lateral ventricle with extension to the third ventricle was the main adopted surgical route. GTR was achieved in all cases without postoperative complications nor postoperative new onset neurological deficits. No patients required ventricular shunting procedures for hydrocephalus during the surveillance period. Follow-up ranged from 43 to 54 months. Ki67-MIB1 values ranged from 5% to 10%. No patients underwent adjuvant radiotherapy. Our findings suggest that GTR alone may be sufficient even in patients with moderately elevated Ki-67. A tailored, risk-adapted postoperative strategy is recommended rather than routine adjuvant treatment.

## Introduction

Several neoplastic [1], vascular [2] and infectious diseases, may primarily or secondarily involve cerebral ventricular chambers, representing sometimes a diagnostic and therapeutic challenge with significant variability between adult and pediatric populations. This issue includes central neurocytoma (CN), a very rare neuronal tumor (0.3% of all intracranial tumors) [3, 4], arising from neuronal cells of septum pellucidum and subependymal cells of lateral ventricles, therefore involving the supratentorial ventricular system. It is classified as grade 2 neuronal and mixed neuroglial tumor according to the 2021 World Health Organization (WHO) classification [5], and mainly affects young adults (mean patient’s age 28.5 years) [5].

Since its first description, surgical resection has remained the cornerstone of treatment, aiming gross total resection (GTR), which is associated with excellent recurrence-free and overall-survival [6–8]. However, this goal is not always achievable, ranging from 48% to 70% of cases [6, 8–11], because of the intrinsic nature of the lesion, which is richly vascularized, and its intimate anatomical relationship with surrounding highly functional nervous structures (e.g. the thalamus, hypothalamus, fornix). Therefore, subtotal resection (STR) followed by radiation therapy is an alternative option of treatment [6, 10, 12].

Nevertheless, the role of adjuvant radiotherapy (aRT) is still controversial, even after GTR, and particularly in cases with increased proliferative activity, as well as the definition of “atypical” neurocytoma, often based on Ki-67 thresholds, that remains inconsistent across studies [8, 11]. Consequently, postoperative management varies widely among institutions.

In this scenario, by reviewing a mono-institutional case series of patients with CN undergone surgical resection, we analyze and discuss the main risk factors for recurrence and which affect the treatment strategy and patient’s outcome, including: (i) the extent of resection (EOR) and surgical tenets to increase the safety of GTR; (ii) the role of Ki67 values in the decision-making process of treatment after surgical resection; (iii) the role of adjuvant radiotherapy.

## Methods

Medical record data of patients with confirmed diagnosis of central neurocytoma who underwent surgical resection at Department of Neurosurgery of “Ospedale del Mare” in Naples – Italy, between January and December 2022, were retrospectively reviewed. Inclusion criteria were adult patients (> 18 years old), minimum follow up 42 months, complete demographic (sex and age), clinical (presenting symptoms), neuroradiological (location and size) and pathological (ki67-MIB1) data, type of management (surgical approach, extent of resection, adjuvant treatments), complications and outcome, time to recurrence, pre- and postoperative hydrocephalus and shunt dependency, follow-up duration.

### Study design

All patients underwent neuro-ophthalmological assessment at admission. All surgical procedures were performed with assistance of intraoperative neuronavigation system, neurophysiological monitoring and ultrasound aspirator. Brain computed tomography (CT) scan was always performed the day after surgery. Postoperative contrast-enhancing magnetic resonance imaging (MRI) of the brain was performed 3 months after surgery in all patients.

## Results

### Case series

#### Case 1

**Fig. 1:**
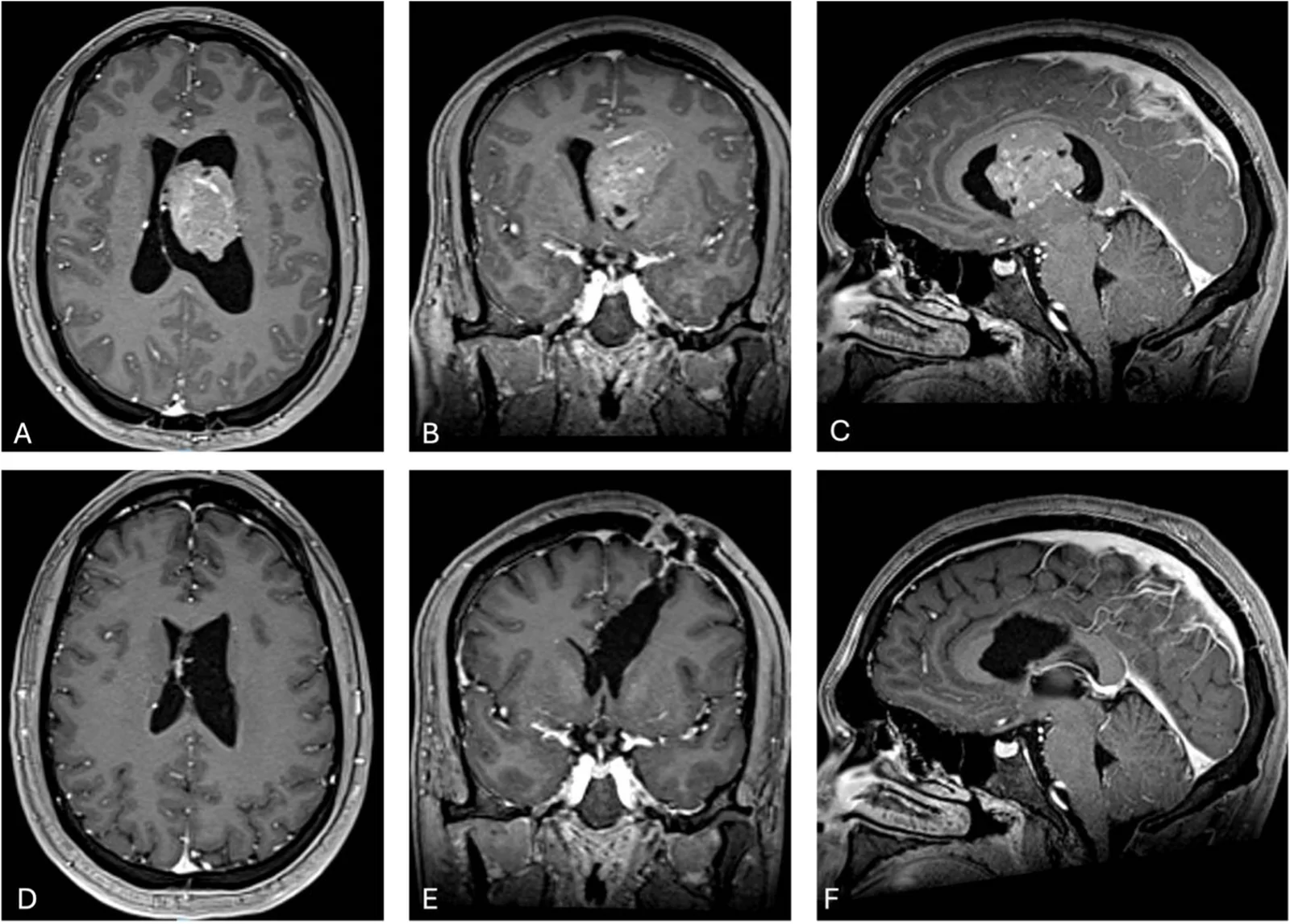
Preoperative brain MRI, axial (**A**), coronal (**B**) and sagittal (**C**) sequences. Solid and highly vascularized lesion, with regular margins, heterogeneous signal intensity and mild, non-uniform enhancement, was evident in the left ventricle. Intralesional microvascular flow voids were also observed. Postoperative brain MRI at last follow-up, axial (**A**), coronal (**B**) and sagittal (**C**) sequences. Gross-total lesion removal and no regrowth were evident

A twenty-four-years old man with history of drug-resistant chronic headache, complained recent onset of diplopia and visual deficit. Following an episode of loss of consciousness, he performed a contrast-enhancing MRI of the brain (Fig. 1A-C) that revealed, in the left ventricle, a solid lesion, with regular margins, characterized by heterogeneous signal intensity and mild, non-uniform enhancement (maximum diameters 46 × 28 mm). Intralesional microvascular flow voids and increased rCBV and rCBV ratio at the lesion site were also observed. The left internal cerebral vein was compressed and displaced caudally, while the ipsilateral superior thalamostriate vein was compressed and displaced superiorly.

Neurological examination at admission was negative. In supine position, patient underwent left frontal transcortical approach to ipsilateral lateral ventricle. The lesion appeared soft in consistency, highly vascularized, without infiltrating the surrounding parenchyma, but strongly adherent to the choroid plexus and the lateral wall of the ventricle. Initial debulking was performed with ultrasound aspirator, then the lesion was deafferented through bipolar coagulation of its implant on the lateral wall ventricle, including coagulation of choroid plexus, and finally detached and completely removed in piecemeal fashion.

The outcome was without clinical and surgical complications and patient was discharged in 5th postoperative day.

Histopathological examination was in favor of atypical central neurocytoma WHO grade 2, with proliferation index Ki67-MIB1 5–6%. Adjuvant radiotherapy was not performed. At last clinical and neuroradiological follow-up, 50 months after procedure, patient was free from pathology (Fig. 1D-F) and in good neurological conditions.

#### Case 2

**Fig. 2:**
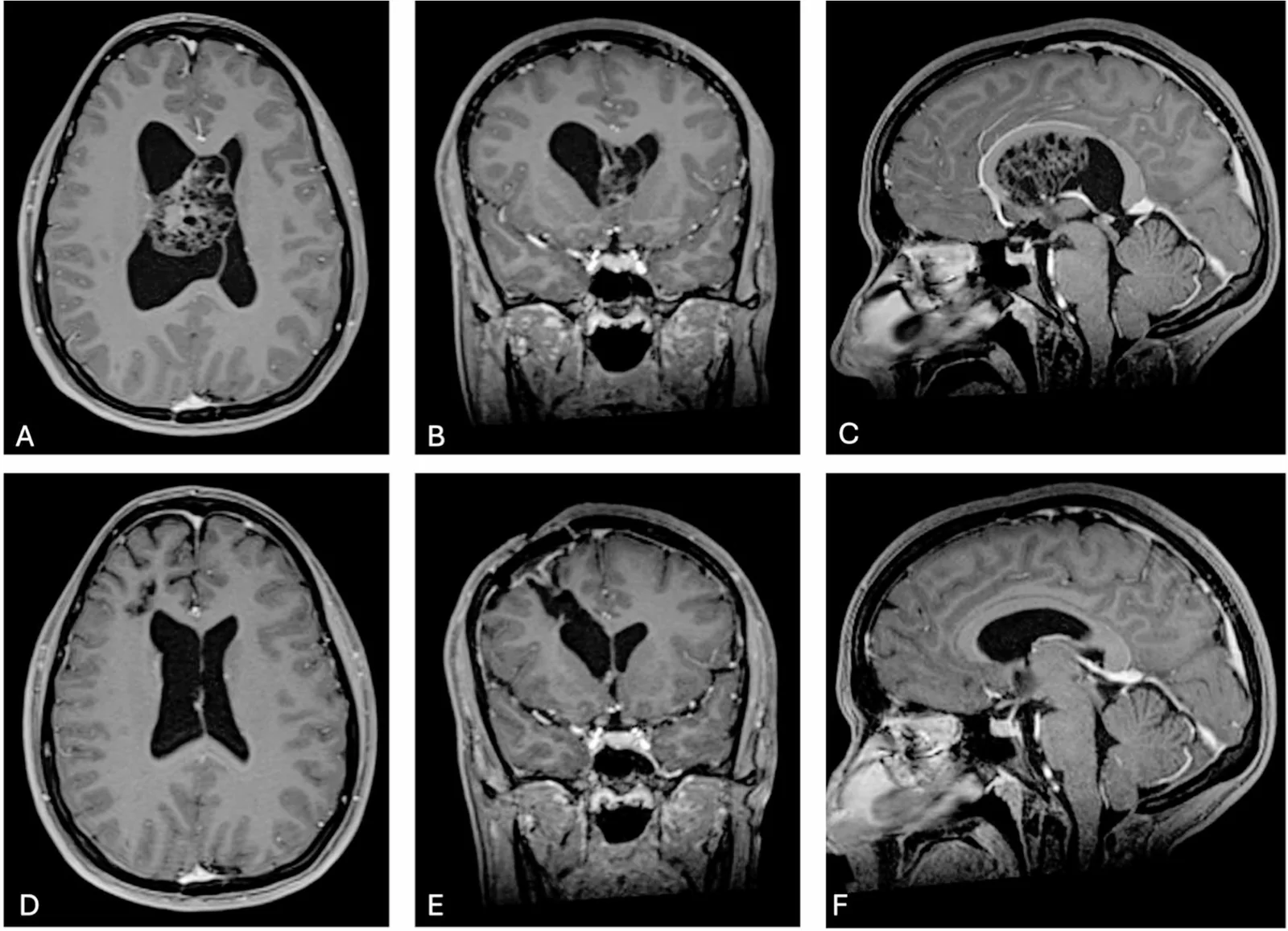
Preoperative brain MRI, axial (**A**), coronal (**B**) and sagittal (**C**) sequences. The lesion in the right lateral ventricle caused enlargement of the ventricle and contralateral shift of the septum pellucidum, as well as lateral deviation and compression of the fornix columns on the left side, along with marked indentation and compression of the left lateral ventricle with initial ependymal transudation. The corpus callosum appeared compressed and thinned. Postoperative brain MRI at last follow-up, axial (**A**), coronal (**B**) and sagittal (**C**) sequences. Gross-total lesion removal and no regrowth were evident. Perioptic sheaths was markedly reduced

A twenty-two-years old female with history of chronic headache and visual deficit getting progressively worse was observed. A contrast-enhancing MRI of the brain (Fig. 2A-C) revealed a large (39 × 42 × 36 mm) right intraventricular lesion, extending caudally to the foramen of Monro and third ventricle, characterized by highly heterogeneous signal and contrast-enhancement in the predominantly parenchymal components. Smooth distension of the perioptic sheaths but with the optic nerve signal preserved were also detected. Perfusion study showed a minimal increase in rCBV values.

Neuro-ophthalmological examination at admission confirmed bilateral visual deficit and papilledema. In supine position, patient underwent right frontal transcortical approach to ipsilateral lateral ventricle. A grayish lesion, friable in consistency, moderately vascularized, and infiltrating the surrounding parenchyma was observed. The tumor was completely removed in piecemeal resection.

The outcome was without clinical and surgical complications and patient was discharged in 7th postoperative day.

Histopathological examination was in favor of atypical central neurocytoma WHO grade 2, with proliferation index Ki67-MIB1 8–10%. Adjuvant radiotherapy was not performed. At last clinical and neuroradiological follow-up, 45 months after procedure, patient was in good neurological conditions and no recurrence was detected (Fig. 2D-F).

#### Case 3

**Fig. 3:**
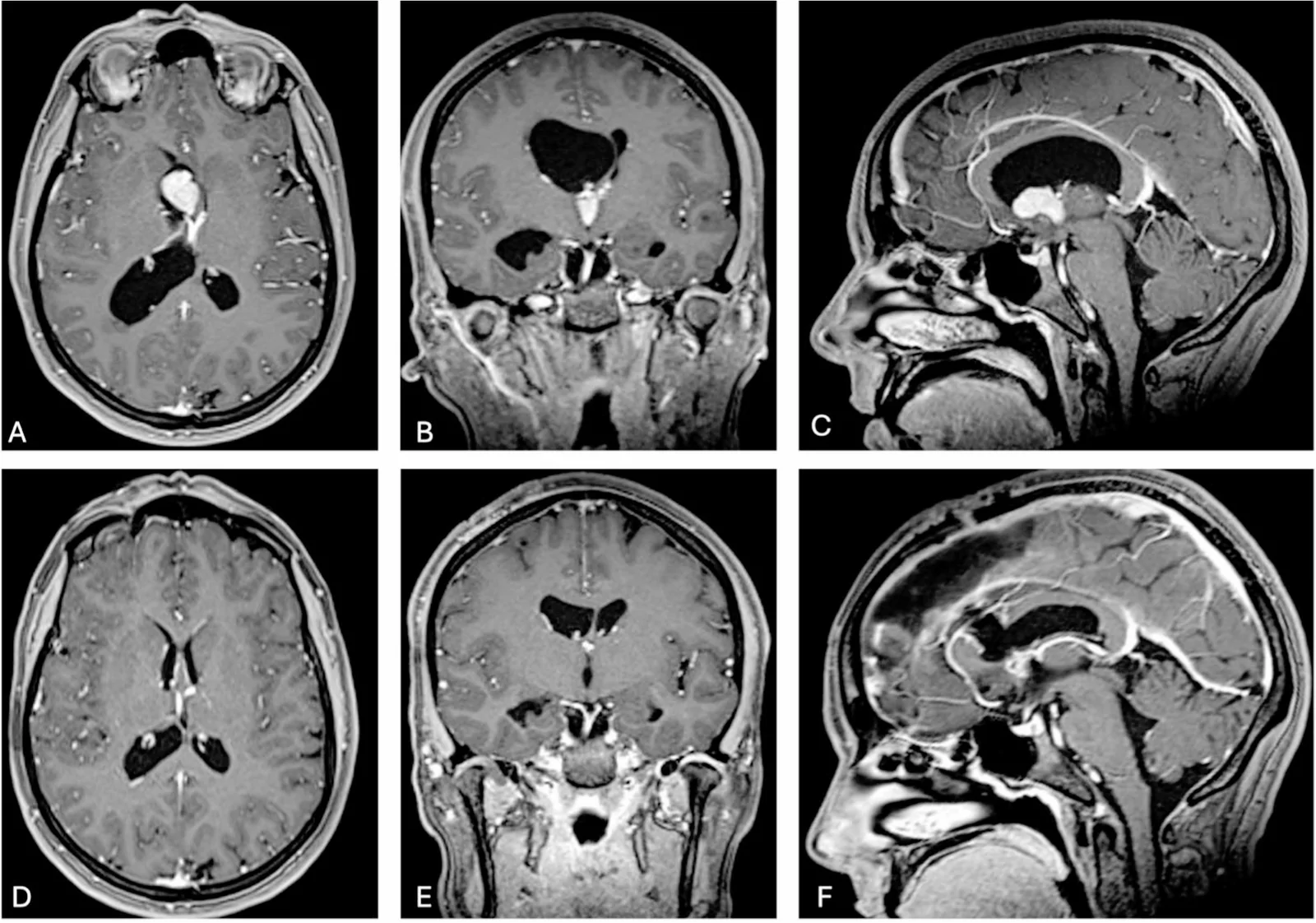
Preoperative brain MRI, axial (**A**), coronal (**B**) and sagittal (**C**) sequences. The lesion in the right lateral ventricle and involving the third ventricle, caused an expansive effect on the adjacent right ventricular middle cell with contralateral displacement of the pellucid septum. Signs of transependymal cerebrospinal fluid transudation were also present. Postoperative brain MRI at last follow-up, axial (**A**), coronal (**B**) and sagittal (**C**) sequences. Gross-total lesion removal and no regrowth were evident

A twenty-nine-years old female complained for about 3 months exertional headaches responsive to medical treatment, associated to episodes of vomiting—primarily in the morning—and visual scotomas in the left eye. She performed a contrast-enhancing MRI of the brain (Fig. 3A-C) that showed, within the right ventricle, also involving the corresponding foramen of Monro and extending marginally caudally to the anterior portion of the third ventricle, a solid lesion with lobulated margins, measuring approximately 21 × 18 mm, showing intense and homogeneous contrast enhancement.

Neuro-ophthalmological examination at admission confirmed visual deficit. In supine position, patient underwent right interhemispheric transcallosal approach to the third ventricle. A pinkish lesion, soft in consistency, moderately vascularized, and firmly attached to the medial wall of the lateral ventricle was observed. In piecemeal resection, alternating ultrasound aspirator and bipolar coagulation, the lesion was completely resected.

The outcome was without clinical and surgical complications and patient was discharged in 6th postoperative day.

Histopathological examination was in favor of atypical central neurocytoma WHO grade 2, with proliferation index Ki67-MIB1 9%. Adjuvant radiotherapy was not performed. At last clinical and neuroradiological follow-up, 48 months after procedure, patient referred improved neurological conditions and no recurrence was detected (Fig. 3D-F).

#### Case 4

**Fig. 4:**
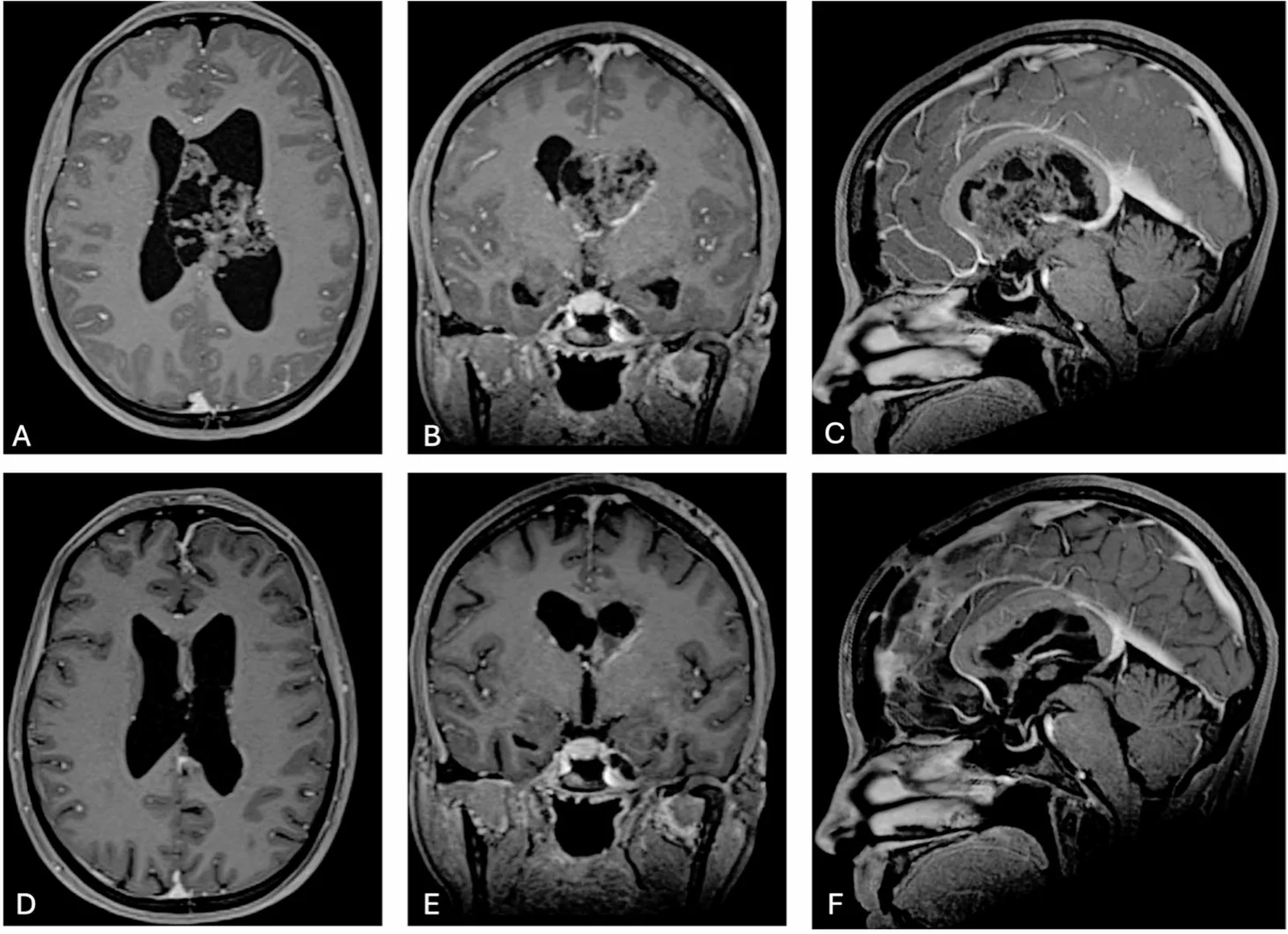
Preoperative brain MRI, axial (**A**), coronal (**B**) and sagittal (**C**) sequences. there is dysmorphism and a marked increase in the size of the supratentorial ventricular system, particularly the left lateral ventricle at the level of the trigone-occipital horn, with a rightward deviation of the septum pellucidum. No signs of ongoing transependymal cerebrospinal fluid transudation are evident at present. The fourth ventricle is in its normal position and regular in shape. The optic nerves have a tortuous course with mild distension of the perineural cerebrospinal fluid space on both sides. The pituitary parenchyma is atrophied. Postoperative brain MRI at last follow-up, axial (**A**), coronal (**B**) and sagittal (**C**) sequences. Gross-total lesion removal and no regrowth can be appreciated

As part of further diagnostic evaluation for recurrent episodes of dysmenorrhea, a twenty-six years old female underwent a contrast-enhancing MRI of the brain (Fig. 4A-C) that showed a coarse, expansive intraventricular lesion (59 × 39 mm), primarily located at the level of the left ventricular middle cell with close adhesion to the septum pellucidum, and at the level of the third ventricle, with heterogeneous appearance due to the coexistence of solid and cystic portions and multiple foci of possible calcific nature within the lesion; mild, diffuse signs of enhancement were observed in the solid portions, accompanied by increased rCBV values on perfusion imaging.

Neurological examination at admission was normal. In supine position, patient underwent left frontal transcortical approach to the ipsilateral ventricle. The lesion was completely removed by dissecting the portion that appeared to be adherent to the floor and the ventricular septum and that extended through the middle cell from the anterior horn to the posterior horn and through the foramen of Monro toward the third ventricle.

The postoperative course was uneventful and patient was discharged in 5th postoperative day.

Histopathological examination was in favor of atypical central neurocytoma WHO grade 2, with proliferation index Ki67-MIB1 9%. Adjuvant radiotherapy was not performed. At last clinical and neuroradiological follow-up, 54 months after procedure, patient was in good neurological conditions and no recurrence was detected (Fig. 4D-F).

#### Case 5

**Fig. 5:**
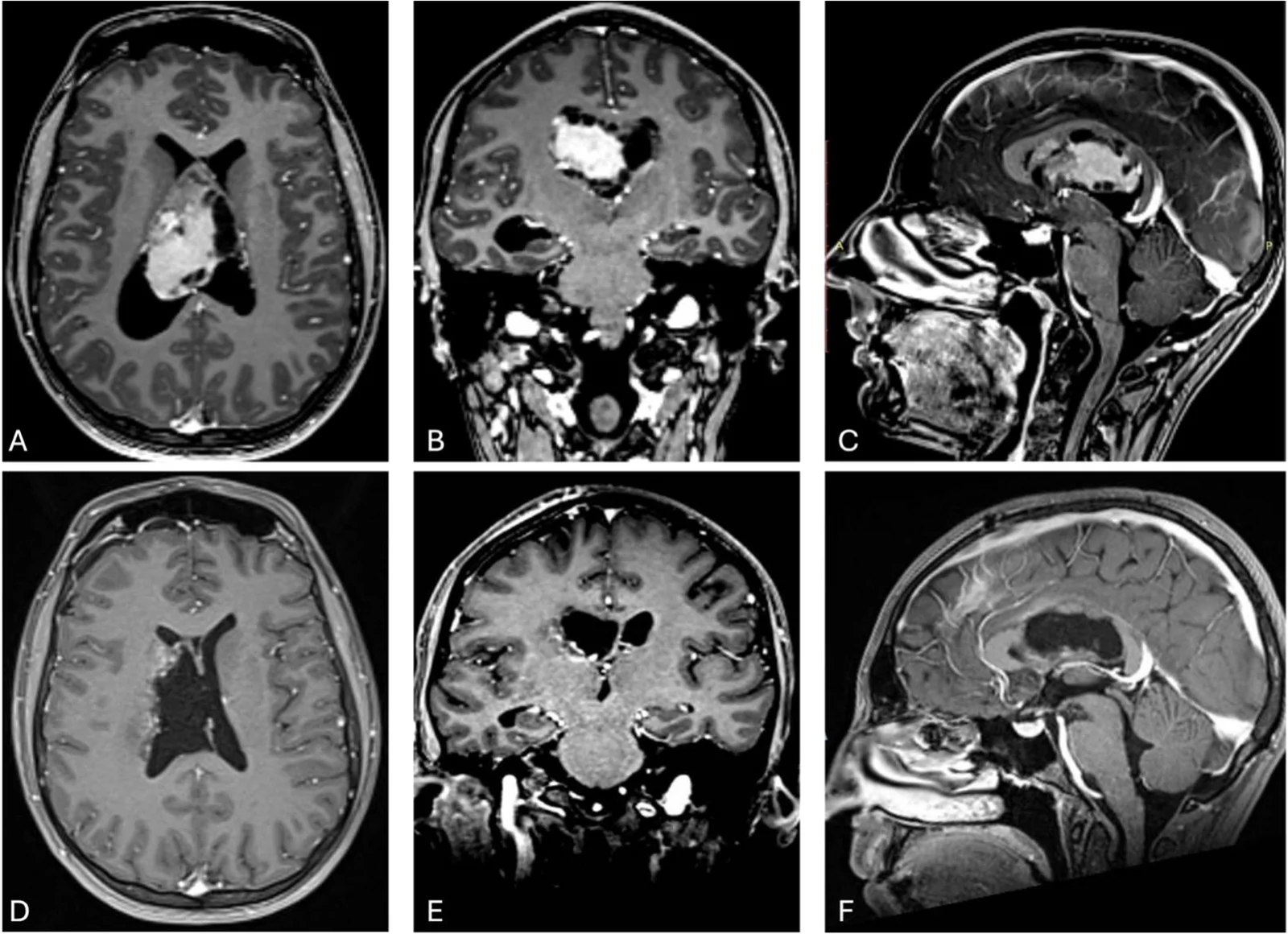
Preoperative brain MRI, axial (**A**), coronal (**B**) and sagittal (**C**) sequences. The lesion in the right lateral ventricle caused enlargement of the ventricle and contralateral shift of the septum pellucidum, as well as lateral deviation and compression of the fornix columns on the left side. Postoperative brain MRI at last follow-up, axial (**A**), coronal (**B**) and sagittal (**C**) sequences. Gross-total lesion removal and no regrowth were evident

A nineteen-years old female for the recent onset of diplopia, as further diagnostic investigation, performed a contrast-enhancing MRI of the brain (Fig. 5A-C) that showed a large (48 × 36 mm) right intraventricular lesion, extending caudally to the foramen of Monro and third ventricle, featuring heterogeneous signal and contrast-enhancement. Perfusion study showed a minimal increase in rCBV values.

Neurological examination at admission was normal while ophthalmological assessment confirmed the visual disturbance. In supine position, patient underwent right frontal transcortical transventricular approach to the ipsilateral lateral ventricle. A grayish lesion, soft/friable in consistency, moderately vascularized, and firmly attached to the ventricle’s lateral wall was observed. Through ultrasound aspirator and bipolar coagulation, the lesion was completely resected.

The postoperative course was regular, without complications, and patient was discharged in 7th postoperative day.

Histopathological examination was in favor of atypical central neurocytoma WHO grade 2, with proliferation index Ki67-MIB1 8–10%. Adjuvant radiotherapy was not performed. At last clinical and neuroradiological follow-up, 43 months after procedure, patient was in good neurological conditions and no recurrence was detected (Fig. 5D-F).

#### Summary

Personal case series included 4 women and 1 man, with mean age 24 years old. Main presenting symptoms were headache and visual deficit. Lesions always involved lateral ventricles, with extension to the third ventricle in all but one cases. Four patients underwent transcortical approach to the ipsilateral lateral ventricle with extension to the third ventricle. In one case, an interhemispheric transcallosal approach was adopted. Gross-total tumor resection was achieved in all cases without postoperative complications. No patients required ventricular shunting procedures for hydrocephalus during the surveillance period. Follow-up ranged from 43 to 54 months. Ki67-MIB1 values ranged from 5% to 10%. No patients underwent aRT.

All patients’ data are summarized in Table 1.

**Table 1:** Demographic, neuroradiological, surgical and histopathological data of monoinstitutional case series

Cases	Age/Sex	Tumor Location	Surgical Approach	EOR	Ventricular shunting	Follow up (months)	Ki67 values	aRT
1	24 M	Left lateral ventricle	L. Transcortical trans ventricular	GTR	No	50	5–6%	No
2	22 F	Right lateral ventricle + 3rd	R. Transcortical trans ventricular	GTR	No	45	8–10%	No
3	29 F	Right lateral ventricle + 3rd	Interhemispheric Transcallosal	GTR	No	48	9%	No
4	26 F	Left lateral ventricle + 3rd	L. Transcortical trans ventricular	GTR	No	54	9%	No
5	19 F	Right lateral ventricle + 3rd	R. Transcortical trans ventricular	GTR	No	43	8–10%	No

*EOR* Extent of Resection, *aRT* adjuvant Radiation Therapy. *M* Male, *F* Female, *L.* Left, *R* Right

## Discussion

The primary end-point of our study was to assess the role of extent of resection which remains paramount.

The rate of GTR in CNs reported in the pertinent literature, in fact, ranges from 48% to 70% [6, 8–10, 13, 14] and the related recurrence rate is between 7% and 20%, consistently lower than after subtotal resection [6, 8, 10, 15] where it is up to 33% [9]. Recurrence is less about “biological aggressiveness alone” and more about subtle surgical residual disease in ventricular corners and attachment zones. Therefore, as the extent of resection is the only well-demonstrated risk factor of recurrence, here we analyze the surgical aspects that help ensure the safety of the procedure, identifying ten key points.

I) The goal of surgery is to achieve true “anatomical” GTR, not just macroscopic GTR. Recurrence most often originates from microscopic residual tumor at the septal, fornicial, or choroidal attachment zones, even when the cavity appears clean. Therefore, complete ventricular wall inspection plus coagulation of tumor base is more important than bulk removal alone.
II) Optimal surgical corridor selection is fundamental to minimize blind angles. Poor trajectory selection is a hidden cause of “hidden residual tumor” in ventricular recesses. Transcortical-transventricular approach is optimal for large tumors affecting lateral and dilated ventricles. It is important to choose entry point closest to maximal tumor bulk and avoid narrow working angles that prevent visualization of the contralateral ventricular wall. Transcallosal approach is optimal for midline lesions with bilateral extension. In these cases, interforniceal dissection must be minimized, and fornices must be preserved to reduce cognitive morbidity without compromising exposure.
III) Systematic ventricular wall sweep. As recurrence often originates from small remnants adherent to the ependyma rather than bulk tumor, after tumor debulking, meticolous inspection of septum pellucidum, foramen of Monro, choroid plexus insertion, ventricular trigones, through angled endoscope or microscope tilt is strongly suggested.
IV) Aggressive management of tumor attachment (key recurrence driver). Most central neurocytomas arise from septal region, choroid plexus or ventricular wall interface. Therefore, in terms of technical strategy we suggest to coagulate tumor base thoroughly, gently shave the ependymal attachment plane and avoid leaving “thin carpet-like remnants”. In this setting, however, it is worth to remember the importance of a balance between aggressive coagulation - which reduces recurrence - and excessive deep coagulation – which could account for venous or fornicial injury.
V) Endoscopic assistance. The adjunct of endoscope, especially if angled, allows the visualization of hidden recesses, the inspection behind septal folds and confirmation of complete tumor resection. It is especially useful for lesions with trigonal extension and for contralateral ventricular inspection.
VI) Vascular preservation without compromising oncological radicality. Feeding vessels typically arise from choroidal arteries and/or pericallosal branches (via septal region). In this scenario, early devascularization reduces intraoperative bleeding and improves en bloc resection; nevertheless, coagulation must be done distal-to-proximal to avoid premature tumor swelling.
VII) En bloc vs. piecemeal resection. Piecemeal resection is usually necessary, but central core decompression should precede wall dissection and avoid leaving “fragmented caps” adherent to ventricular lining. In fact, recurrence risk increases when residual thin fragments remain attached to the ependyma in trigonal or septal areas.
VIII) Hemostasis technique matters more than usually appreciated. Excessive bipolar coagulation on ventricular walls can obscure residual tumor and create “false clean field”. In this setting, preferred strategy includes gentle irrigation, minimal coagulation after tumor removal and reassessment of all walls after hemostasis.
IX) Intraoperative imaging. Useful tools include neuronavigation system for trajectory optimization, intraoperative ultrasound (in selected cases), endoscopic confirmation in deep blind corners. These are helpfull to reduce “false GTR”.
X) Conceptual takeaway. “Recurrence of central neurocytoma is less a failure of extension of resection and more a failure of ventricular surface inspection and attachment-site management.”

The secondary end-point was to discuss the role of Ki67 in the decision-making process of treatment after surgical resection.

Several literature reports suggest that values of Ki67 > 2–3% are associated to a more aggressive tumor behavior and higher recurrence risk; therefore, they consider the values of these markers for guiding the postoperative management [6, 10, 11, 16]. However, it is worth considering some points.

First, it is worth to remember that Ki67 proliferation index and mitotic count are limited by poor reproducibility and considerable interlaboratory variability. Fo the same reason, despite the use of this marker to assess the degree of malignancy has been repeatedly proposed also for other tumors of the central nervous system, the WHO has never implemented it into the grading system.

In addition, its clinical role remains controversial, with inconsistent thresholds reported across studies [12]. Notably, in small case series, although small, no recurrences were observed despite Ki-67 values up to 10% and no aRT. This finding, first questions the reliability of commonly used cutoffs (2–3%) for defining “atypical” tumors - thus Ki67 thresholds may be overestimated -, and then suggests that proliferative index alone may not justify adjuvant treatment.

In this scenario, it is worth to remember the role still unclear of molecular biomarkers in CN. Considering the last WHO classification of central nervous system tumors [5], conversely from other lesions, for which molecular profiling has become integral part into diagnosis, prognosis, and tailored treatment strategy, CN continues to be managed based on histopathological features and the extent of surgical resection.

Finally, we analyze the role of adjuvant radiotherapy.

While it is well-demonstrated that aRT is associated with longer PFS in STR patients [8, 10, 13, 14, 17, 18], there is not unanimous consent in the pertinent literature about its benefit in terms of PFS in GTR patients: according to Krech et al. [8], in fact, aRT does not prolong PFS in this group of patient, conversely Wee et al. [11] argue that it may improve PFS, especially in patients with elevated Ki67 index (> 2%).

Given the significant side effects of radiation therapy, such as cognitive decline, radiation necrosis, or radiation-induced malignancy, it is crucial to weigh risks and benefits carefully, especially in young patients. The absence of clear survival benefit in the literature combined with our findings suggests that routine radiotherapy after GTR may represent a not justified overtreatment. Therefore, we suggest to administer RT in recurrent or subtotal resected CN, whereas in cases of GTR we believe that a more conservative, individualized approach, based on radiological follow-up may be appropriate.

In conclusion, as patient and tumor’s features affect the strategy of treatment, as for other benign lesions in critical locations, like spheno-orbital meningiomas, a balance between a more aggressive versus more conservative approach – and thus between progression-free survival and quality of life - should be taken into account: aggressive surgery aiming gross total resection prolongs PFS, avoids side effects of aRT, but with related surgical risks for the surrounding neurovascular structures; on the other side, a more conservative surgery, planning aRT, but with the potential side effects of radiation treatment. Finally, especially in young patients, even multiple surgeries for symptomatic recurrences over the lifetime can be considered an option of treatment. In this setting, a recent multicenter study [19] on CN patients treated by surgical resection showed generally favorable long-term functional outcomes after surgical treatment, with high long-term health-related quality of life scores. However, cognitive outcomes remained severely affected with only one-third of patients achieving favorable results.

Given the rarity of central neurocytoma and the absence of universally accepted treatment guidelines, postoperative management should ideally be discussed within a multidisciplinary neuro-oncology board, involving neurosurgeons, neuroradiologists, neuropathologists, radiation oncologists and neuro-oncologists to individualize patient care. In addition, future multicenter collaborative studies and prospective registries are necessary to improve statistical robustness and provide stronger evidence regarding prognostic factors, the biological significance of Ki67-MIB1, and indications for adjuvant treatment.

## Conclusion

Our findings support a cautious approach toward avoiding upfront radiotherapy in selected patients with atypical central neurocytomas who have undergone gross-total, even in the presence of moderately elevated proliferative indices. However, these patients require close clinical and radiological surveillance over many years because late recurrences may occur. Our findings suggest that Ki-67 alone should not dictate postoperative management after gross total resection. We consider administration of RT in recurrent or subtotal resected CN. A tailored, risk-adapted postoperative strategy is recommended rather than routine adjuvant treatment. Further studies, with large case series and/or multicenter studies, are needed to better identify prognostic factors, the implication of Ki67-MIB1 values, and the role and indications of adjuvant treatment.

## Limitations

The retrospective nature of the study is the first limit of the study. We agree that the sample size is limited. However, central neurocytoma is a rare tumor, and single-institution series are inherently small. Another strong limitation is that epigenetic landscape of CN was not studied.

Importantly, the homogeneity of our cohort (all GTR, no adjuvant therapy) strengthens the internal consistency of our observations.

## Data Availability

No datasets were generated or analysed during the current study.
